# Atezolizumab-Induced Sarcoidosis-Like Reaction in a Patient with Metastatic Breast Cancer

**DOI:** 10.1155/2022/2709062

**Published:** 2022-01-27

**Authors:** Akira Tsunoda, Toshiro Mizuno, Shohei Iida, Katsunori Uchida, Masako Yamashita, Koshi Sukeno, Hiroki Oka, Yasutaka Tono, Mikiya Ishihara, Kanako Saito, Satoshi Tamaru, Keiichi Yamanaka, Isao Tawara

**Affiliations:** ^1^Department of Medical Oncology, Mie University Hospital, 2-174 Edobashi, Tsu, Mie Prefecture 514-8507, Japan; ^2^Department of Dermatology, Mie University Graduate School of Medicine, 2-174 Edobashi, Tsu, Mie Prefecture 514-8507, Japan; ^3^Department of Oncologic Pathology, Mie University Graduate School of Medicine, 2-174 Edobashi, Tsu, Mie Prefecture 514-8507, Japan; ^4^Department of Breast Surgery, Mie Prefectural General Medical Center, Ohji-Hinaga 5450-132, Yokkaichi, Mie Prefecture 510-8561, Japan

## Abstract

Tumor-related sarcoidosis-like reactions (SLR) have been reported with the use of immune checkpoint inhibitors (ICIs). We report a case of 50-year-old woman who observed an enlarged lymph node in the right hilar region and the appearance of a subcutaneous mass in the extremities during chemotherapy with atezolizumab plus nab-paclitaxel for metastatic triple-negative breast cancer (TNBC). Skin biopsy revealed the formation of epithelioid granulation species with the Langhans giant cell. After discontinuing atezolizumab in the treatment procedure, the hilar lymph nodes and the subcutaneous mass were reduced. A pathological examination was effective in differentiating tumor exacerbation from SLR. Owing to limited information on ICI-related SLR in breast cancer, future studies are recommended to properly manage immune-related adverse effects during cancer treatment.

## 1. Introduction

Triple-negative breast cancer (TNBC) constitutes 15–20% of cases of breast cancer and is characterized by the absence of estrogen receptors (ER), progesterone receptors (PgR), and overexpression or gene amplification of human epidermal growth factor receptor type 2 (HER2) on the surface of cancer cells [1, 2]. Recently, the results of IMpassion130 phase 3 clinical trial reported superiority in progression-free survival of the anti-programmed death ligand 1 (PD-L1) monoclonal antibody atezolizumab plus nab-paclitaxel over placebo plus nab-paclitaxel as a first-line treatment for patients with advanced or metastatic TNBC [2]. While therapeutic effectiveness of immune checkpoint inhibitors (ICIs) has been demonstrated, management of immune-related adverse events (irAEs) during treatment remains a concern. Reportedly, irAEs occur in various parts of the body, causing endocrine and neurological disorders. One rare irAE is sarcoidosis-like reactions (SLR). Although tumor-related SLRs are sometimes reported with the extensive use of ICIs, their pathogenesis and etiology are not fully understood [3]. We report a case of SLR during atezolizumab plus nab-paclitaxel ICI therapy for metastatic TNBC, with a literature review on the mechanism of the said irAEs.

## 2. Case Report

**Figure 1:**
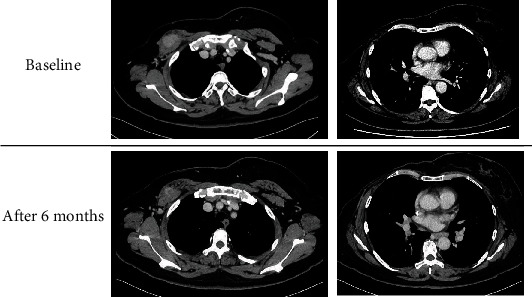
At baseline, computed tomography (CT) shows the appearance of the right subpectoral lymph node metastasis (3.6 cm diameter). After 6 months, CT showed further reduction of that lesion, while the right hilar lymph nodes are enlarged.

A 50-year-old woman was diagnosed with breast cancer and underwent right mastectomy with axillary lymph node dissection. The pathological findings revealed invasive ductal carcinoma, pT2 (3.0 cm) pN1a (3/22) cM0, and pStageIIB. The immunostaining confirmed ER negative, PgR negative, and HER2 2+ (FISH: no amplification). She had no history of smoking, and none of her family members who lived with her were smokers. Her past medical history was unremarkable. Adjuvant chemotherapy was performed with 4 cycles of epirubicin 90 mg/m^2^ plus cyclophosphamide 600 mg/m^2^ on day 1 every 3 weeks, followed by 12 cycles of paclitaxel 80 mg/m^2^ on day 1 every week [4, 5]. Two years after the surgery, an enlarged lymph node (*φ* 2.8 cm) in the right pectoralis major muscle was observed (Figure 1). Cytology showed adenocarcinoma, indicating recurrence of breast cancer. The programmed death ligand 1 (PD-L1) expression with SP 142 was positive (IC: 3) in the resected tissue. As a first-line treatment, the patient received atezolizumab (840 mg) per body on days 1 and 15 plus nab-paclitaxel 100 mg/m^2^ on days 1, 8, and 15 every 28-day cycle [2]. With the treatment, the lymph node metastases reduced.

**Figure 2:**
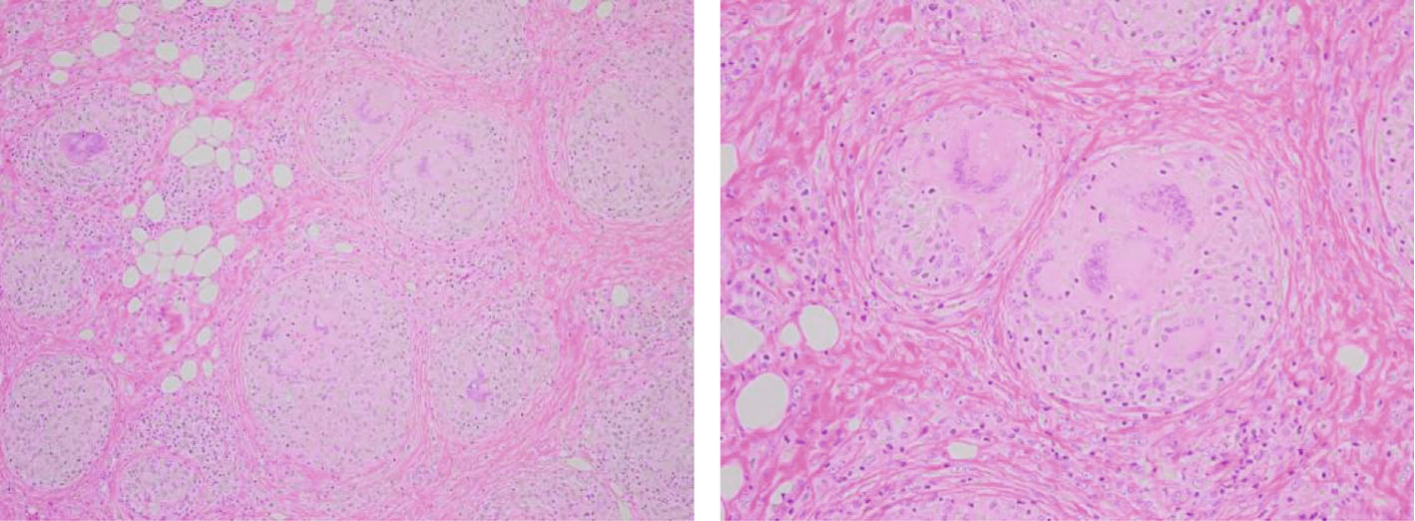
Exploration of subcutaneous mass in the lower extremities. Hematoxylin and eosin stain shows epithelioid cell granuloma with Langhans giant cells. There are no necrotic tissue and no similarity to surgical specimens of breast cancer.

After 5 cycles of atezolizumab plus nab-paclitaxel, an enlarged lymph node in the right hilar region, and appearance of a subcutaneous mass in the extremities were observed (Figure 1). Her vitals were stable, and the blood test results showed no abnormalities (Table 1). Skin biopsy was then performed on the subcutaneous mass, which showed formation of epithelioid granulation species with Langhans giant cells (Figure 2). The skin tissue culture test did not detect the presence of any organism that could be associated with the appearance of the nodules.

From these findings, we determined that SLR resulted from the use of atezolizumab. Atezolizumab was discontinued for the treatment of SLRs, while nab-paclitaxel monotherapy was continued. After the change in treatment, there was a reduction in the size of the hilar lymph nodes and that of the subcutaneous mass. However, it was thought that total 8 cycles of nab-paclitaxel became refractory due to the enlargement of the metastatic lesion just below the right pectoral major muscle. Thereafter, chemotherapy such as eribulin, capecitabine, and irinotecan for three to four cycles each depending on metastatic TNBC progression has been administered [6–8]. The patient is currently undergoing carboplatin plus gemcitabine, while the SLR-related lesions have remained reduced to date [9].

## 3. Discussion

The use of immunotherapy in the field of oncology has revolutionized treatment of cancer. However, with the application of ICIs, incidence of SLR as a type of irAE is also increasing. According to a previous report, SLR is associated with 4.4% of malignant tumors. In terms of the frequency of malignant tumors by cancer type, it was commonly observed in melanoma, uterine carcinoma, lung cancers, and urethral carcinoma [10]. The time to onset of SLR after initiating ICIs has ranged from 3 weeks to almost 2 years, and there is no obvious ICI dose threshold for the development of ICI-related SLR [10]. The mediastinal lymph node, lung, and skin were common organs involved with ICI-related SLR. Corticosteroid treatment or other antisarcoidosis treatment was not required for all cases of ICI-related SLR [10].

The mechanism of SLR during immunotherapy has been previously described in several studies [10, 11]. It had been shown that cytotoxic T-lymphocyte-associated protein 4 (CTLA-4) blockade enhanced Th17 CD4+ cells in peripheral blood, thus leading to an extended expression of proinflammatory cytokines, such as IL-6 and TNF-*α* [11]. IL-2 secretion by activated T cells is assumed to be involved in the pathogenesis of sarcoidosis [12]. However, information is limited regarding the development of SLR with PD-1 and PD-L1 checkpoint inhibitors than with CTLA-4 [10].

Treatment-related SLR in breast cancer is not only caused by ICIs but also caused by various drugs (Table 2). To date, only one case had been reported by ICI-related SLR. Whether SLR is a characteristic side effect of ICIs in breast cancer requires further investigation through clinical case events. In this case, skin biopsy led to the diagnosis of SLR, which could be appropriately treated with atezolizumab withdrawal alone. Fluorodeoxyglucose-positron emission tomography (FDG-PET) was omitted because the lesions could be adequately identified by CT follow-up (Figure 1).

TNBC behaves more aggressively with earlier relapses and with poorer survival outcomes in comparison to other breast cancer subtypes [13]. If a new lesion is observed in TNBC, it is important to immediately diagnose and assess the pathology to properly discern if changes in current treatment are necessary. If a lesion is found in a site characteristic of SLR, as in the mediastinal lymph nodes, lung, or skin, it is also important to perform a pathological examination to differentiate between tumor exacerbation and SLR.

It has been suggested that the efficacy and safety of cancer immunotherapy may be affected by the concomitant use of drugs such as antimicrobials, proton pump inhibitors, and steroids [14]. In this case, since there was no history of the use of any of the drugs mentioned above, it was inferred that the combination treatment had little effect on immunotherapy and was not associated with the development of SLR.

In conclusion, we herein report a case of a woman with SLR during atezolizumab plus nab-paclitaxel immunotherapy in metastatic TNBC. Further evidence-based studies on ICI-related SLR in breast cancer are recommended to effectively manage immune-related events during cancer treatment.

## Figures and Tables

**Table 1:** Laboratory tests on nodule appearance.

Hematology		
WBC	4.75 × 10^3^	*μ*L
RBC	4.06 × 10^6^	*μ*L
Hb	11.9	g/dL
Ht	37.7	%
Plt	22.5 × 10^4^	/*μ*L
Immunology		
CRP	0.32	mg/dL
*β*-D glucan	<2.8	pg/mL
sIL-2R	691	U/mL
ACE	12.5	U/L
Lysozyme	5.4	MCG/mL
Tuberculosis specific IFN*γ*	Negative	
Blood chemistry		
Total protein	7.6	g/dL
Albumin	4.3	g/dL
AST	19	IU/L
ALT	15	IU/L
LDH	231	IU/L
ALP	206	IU/L
*γ*-GTP	16	IU/L
BUN	13.6	mg/dL
Cre	0.46	mg/dL
Na	142	mEq/L
K	4.0	mEq/L
Cl	104	mEq/L
Ca	9.2	mg/dL
Glucose	92	mg/dL

**Table 2:** Published reports of SLR in patients with breast cancer.

Authors	Age (y)	Subtype			Treatment regimens	References
		ER	PgR	HER2		
Lafon M, et al.	69	0	0	Negative	Nab-paclitaxel plus atezoliumab	[15]
Stoffaës L, et al.	62	95	70	Negative	Palbocicurib plus anastrozole	[16]
Vieira H, et al.	71	90%	95%	Positive	Trasutuzumab plus anastrozole	[17]
Panagiotidis E, et al.	45	Negative	Negative	Positive	Pertuzumab and trastuzumab	[18]
Shin HC, et al.	46	Positive	Positve	Negative	TAM	[19]
. Bhattad PB, et al.	45	Positive	Positive	Negative	TAM	[20]
Martella S, et al.	55	90%	90%	Negative	TAM	[21]
	53	90%	0%	Negative	TAM plus LHRHa	[21]
	53	90%	90%	Negative	TAM plus LHRHa	[21]

ER: estrogen receptors; PgR: progesterone receptors; HER2: human epidermal growth factor type 2; TAM: tamoxifen; LHRHa: luteinizing hormone-releasing hormone agonists.
